# Probing Beyond the Pain Scale: A Rare Case of Cutibacterium Acnes Septic Arthritis

**DOI:** 10.7759/cureus.31864

**Published:** 2022-11-24

**Authors:** Phillip H Keys, George Ishac, Servando T Cuellar, Muhammad A Mushtaq, Sidra Qureshi

**Affiliations:** 1 Department of Internal Medicine, University of Texas Medical Branch at Galveston, Galveston, USA

**Keywords:** vaso occlusive crisis, cutibacterium acnes, sickle cell disease, septic arthritis, sickle cell crisis

## Abstract

Patients with sickle cell disease frequently present to the hospital for pain control secondary to vaso-occlusive crises (VOCs). Diagnostic challenges exist for healthcare providers in distinguishing joint pain secondary to a VOC from an intraarticular infection at initial presentation due to the lack of established clinical markers exclusive to one or the other. We present a 35-year-old female with sickle cell disease and avascular necrosis of bilateral hips and the right shoulder with several previous admissions for VOC pain control complaining of a "different" kind of pain in her shoulder. Treated initially for pain control, our patient was found to be suffering from culture-positive septic arthritis of the shoulder with *Cutibacterium acnes*, a rare source of *de novo* intraarticular infection. This case highlights the importance of incorporating patients’ subjective descriptions of illness into differential diagnosis considerations, notably for those caring for patients with sickle cell disease. This case also establishes* C. acnes *as a rare organism responsible for *de novo* septic arthritis in the setting of sickle cell disease.

## Introduction

Sickle cell disease is a lifelong diagnosis that leaves patients susceptible to many healthcare complications. The most common reason for patients presenting to the hospital are VOCs, also called "pain crises", which cause significant pain for patients as a result of transient focal ischemia [1]. Such infarctions commonly occur at large joints such as hips and shoulders but also affect organs such as the spleen, resulting in decreased clearance of encapsulated organisms [2]. For this reason, infections pose an increased level of danger to those with sickle cell disease.

The clinical pictures of vaso-occlusion and septic arthritis can look similar initially, with patients untreated for a septic joint being in prolonged pain while awaiting an appropriate diagnosis [3]. Our case illustrates that a patient presenting to the hospital with a VOC and a single joint complaint should have septic arthritis as a differential to ensure early diagnosis and treatment. Moreover, we present a rare case of Cutibacterium acnes septic arthritis, reflecting the variety of bacteria able to manifest joint infection in the setting of sickle cell disease.

This case report was previously presented as a poster abstract at the 2022 Southern Hospital Medicine Meeting on October 13, 2022.

## Case presentation

**Figure 1:**
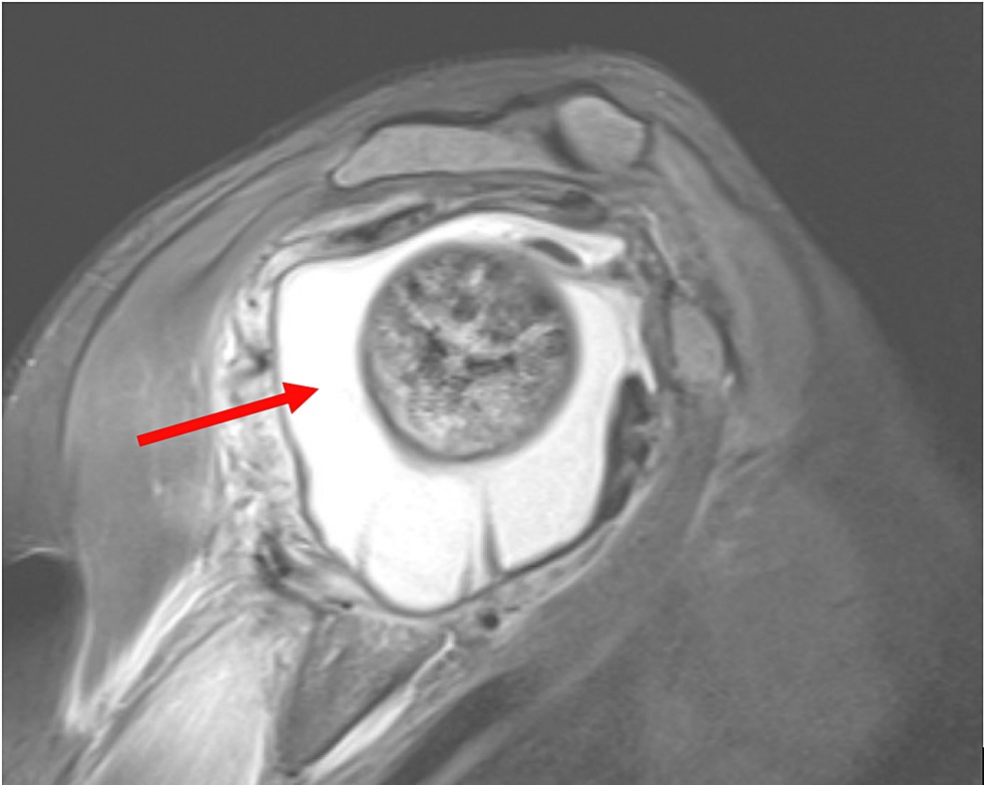
T2 MRI showing a large right glenohumeral joint effusion (red arrow) on day 10 of hospitalization, initiating joint aspiration and culture, and later antibiotic treatment.

The patient is a 35-year-old female with a history of sickle cell disease complicated by bilateral hip avascular necrosis and right shoulder avascular necrosis who presented with two weeks of right shoulder pain. She has had multiple VOCs before but reported the sensation of pain feeling "a bit different" midway through her inpatient stay, described as "in my muscles." She denied any recent viral or bacterial infections and had no history of osteomyelitis or septic arthritis. She denied fever, chills, nausea, vomiting, and trauma and was compliant with her daily hydroxyurea. Due to her frequent hospitalizations, she has a port in her right chest that has been in place for over 10 years. The port is used strictly for hospitalizations. She had not noticed any pain or erythema around the port site. Pertinent labs on admission include a white blood cell (WBC) count of 15.18, hemoglobin of 7.8, reticulocyte count of 7.36, c-reactive protein (CRP) of 19.2, and an erythrocyte sedimentation rate (ESR) of 103. Vital signs were stable, with blood pressure at 101/72, the pulse at 99, the temperature at 99.4 °F, and the respiratory rate at 19 with oxygen saturation at 99% on room air. The physical exam was notable for severe tenderness to palpation on the lateral aspect of the right shoulder joint with no erythema or swelling and limited range of motion due to pain. The right shoulder x-ray did not show an overt fracture. The patient was started on intravenous fluids and an aggressive pain regimen that escalated to a ketamine drip, which provided minimal relief. Due to intractable pain, orthopedic consultants recommended further imaging to rule out joint effusion. A magnetic resonance image (MRI) of the right shoulder showed a large glenohumeral joint effusion (Figure 1).

A joint aspiration yielded gram-positive rods. Two sets of blood cultures drawn from peripheral sites showed no growth. The patient was then started empirically on 750 mg of intravenous vancomycin twice daily and 1 g of ceftriaxone once daily. She began to have pain relief and was weaned off the ketamine drip. The aspiration culture grew 3+ Cutibacterium acnes, and the patient was discharged with a three-week course of 1-gram oral amoxicillin three times daily as the suspicion of C. acnes being a contaminant was low.

## Discussion

Diagnostic differentiation between a VOC and a septic joint traditionally relies on an increase in inflammatory laboratory markers, swelling and erythema of the affected area, a pain response, and, unfortunately, time. The standard of practice upon intake of a patient presenting with evidence of a VOC is to prioritize pain control while ruling out alternative causes of pain (septic arthritis, osteomyelitis, avascular necrosis). Because the clinical pictures of vaso-occlusion and septic arthritis can initially look similar, patients with pain from septic joints are often left untreated for many days while awaiting aspiration and the results of cultures [3]. As no randomized controlled clinical trials inform the treatment of joint infections in the setting of sickle cell disease, general recommendations are to treat patients the same as those without sickle cell disease where joint aspiration dictates antibiotic and/or debridement needs [4]. When evidence of a septic or osteomyelytic joint is evident, empiric treatment with broad-spectrum antibiotics can result in a reduction in the overall incidence of infected joints [5]. However, empiric treatment of patients with vaso-occlusive symptoms also suggestive of an infected joint can lead to antibiotic resistance and increased costs for the patients [6]. Due to the dearth of unambiguous clinical evidence available to a hospitalist physician to distinguish between a VOC and an intraarticular infection at initial presentation, the subjective descriptions of patients with sickle cell disease are important pieces of diagnostic information. Therefore, in the physician’s clinical decision-making process, it is of high importance to prioritize the patient’s subjective pain description as it relates to prior VOC experiences.

Our patient’s glenohumeral joint aspiration contained Cutibacterium acnes. C. acnes is a gram-positive bacillus that commonly makes up human skin flora, especially at the location of the shoulders [7]. Under normal circumstances, C. acnes functions as a commensal organism and skin probiotic, benefiting the host as it has been shown to compete with Staphylococcus epidermidis and stunt the growth of Staphylococcus aureus [8,9]. Nearly half of glenohumeral joints undergoing arthroplasty will return a culture-positive result after the operation [10]. However, only a small percentage of positive cultures reflect true infections, with the most reliable diagnostic criteria being multiple separate positive cultures [10,11]. Interestingly, our patient had a history of glenohumeral avascular necrosis and no prior surgeries. De novo arthritic infections by C. acnes have only been described a handful of times in current literature [12].

As C. acnes commonly makes up human skin flora at the shoulders, the actual rate of intraarticular infections specifically caused by C. acnes colonization remains debated, as it presumably is only a contaminant in many post-operative or post-aspiration cultures [10]. In this case, the initial incision and drainage led to frank purulent drainage, which was later cultured with 3+ C. acnes in one culture fluid and 1+ C. acnes in a second. In a patient without any operative history in her affected shoulder joint, we are led to presume the source of her infection is likely her longstanding implanted venous access port in her right chest.

## Conclusions

In the context of sickle cell disease, the clinical pictures of vaso-occlusion and septic arthritis can appear similar at first, with patients who are untreated for a septic joint experiencing prolonged pain while waiting for an appropriate diagnosis. Our case illustrates that a patient presenting to the hospital with a VOC and a single joint complaint should have septic arthritis as a differential to ensure early diagnosis and treatment, especially in patients with longstanding venous access ports. Moreover, we present a rare case of Cutibacterium acnes septic arthritis, reflecting the variety of bacteria able to manifest joint infection in the setting of sickle cell disease.
